# Clinical efficacy and safety of oral and intravenous vitamin C use in patients with malignant diseases

**DOI:** 10.1007/s00432-021-03759-4

**Published:** 2021-08-17

**Authors:** Catalina Hoppe, Maren Freuding, Jens Büntzel, Karsten Münstedt, Jutta Hübner

**Affiliations:** 1 Klinik für Innere Medizin II, Hämatologie und Internistische Onkologie, Universitätsklinikum Jena, Am Klinikum 1, 07747 Jena, Germany; 2 Klinik für HNO-Erkrankungen, Kopf-Hals-Chirurgie, Interdisziplinäre Palliativstation, Südharz Klinikum Nordhausen, Dr.-Robert-Koch-Straße 39, 99734 Nordhausen, Germany; 3 Gynäkologie und Geburtshilfe, Ortenau Klinikum Offenburg-Kehl, Ebertplatz 12, 77654 Offenburg, Germany

**Keywords:** Vitamin C, Ascorbic acid, Cancer, Complementary medicine, Safety

## Abstract

**Background:**

Vitamin C, also called ascorbic acid, is a water-soluble antioxidant and free radical scavenger. It is required in the body for numerous metabolic functions and is involved in the development of proteins and connective tissue.

**Methods:**

In April 2020, a systematic search was carried out on five electronic databases (Medline, Embase, Cochrane, Cinahl, PsycINFO) to find studies on the use, efficacy and safety of a complementary therapy with vitamin C in oncological patients.

**Results:**

Out of the initial 23,195 search results, 21 studies with 1961 patients were included in this review. Five of the included studies (*n* = 417) were randomized controlled trials (RCTs). The remaining 16 studies belonged to a lower class of evidence. The patients who were treated with vitamin C suffered from various malignant diseases, some in an advanced and palliative stage. Vitamin C was applied intravenously or orally. It was either the only treatment or was combined with chemo- or radiotherapy. Endpoints included the development of the disease-related symptoms, quality of life, mortality, progression-free survival and safety of vitamin C. The studies were of moderate quality and showed either no effect of vitamin C or a positive trend, although this has rarely been statistically proven in group comparisons. No or only slight side effects with both oral and intravenous administration of vitamin C were reported.

**Conclusion:**

Oral intake of vitamin C does not appear to have any effect in patients with malignancies. Data are heterogeneous for intravenous administration. There are no RCTs with statistical group comparisons.

## Introduction

Vitamins and trace elements are among the most frequently used complementary and alternative treatment methods by patients in Germany (Micke et al. 2009). The main reasons for taking these supplements are to strengthen the immune system and reduce side effects (Huebner et al. 2014). With regard to the effects of vitamin C in cancer cells, it is known that it acts as an antioxidant at physiological levels and it could be linked to pro-oxidant effects at pharmacological doses which promote the death of cancer cells (Chen et al. 2005, 2007). Various in vitro studies have already shown that high concentrations of vitamin C led to apoptosis of cancer cells (Chen et al. 2005; Ha et al. 2009; Harakeh et al. 2007; Hong et al. 2007; Kim et al. 2012; Obara and Harasawa 2008). So far, several mechanisms have been described in vitro which give a more detailed insight into the molecular pathways which contribute to the induction of apoptosis (Irimie et al. 2019). An oxidized form of vitamin C, dehydroascorbate, is transported by glucose transporters. In the process of generating energy, cancer cells switch from oxidative phosphorylation to glycolysis. The excess of vitamin C restricts glucose transport and ATP production which leads to energetic crisis and cell death (Pawlowska et al. 2019; Uetaki et al. 2015). In addition, by activating 10–11 translocation proteins and downregulating pluripotency factors, vitamin C can eradicate cancer stem cells (Ramezankhani et al. 2019; Shenoy et al. 2019). In another process, vitamin C promotes the synthesis of collagen-containing anatomical barriers, the breakdown of which leads to metastasis (Boyera et al. 1998). At the same time, it induces degradation of hypoxia-inducible factor, HIF-1, essential for the survival of tumour cells in hypoxic conditions (Pawlowska et al. 2019).

While for apoptosis, the oxidative capacity of ascorbic acid is mandatory, in vivo much lower concentrations are reached which lead to anti-oxidative mechanisms. Accordingly, it is still unclear what effect it has on the efficacy of chemotherapy. Here, dose-dependent effects are also conceivable. In more recent studies, it is assumed that vitamin C reduces the effect of chemotherapy (Heaney et al. 2008; Llobet et al. 2008; Perrone et al. 2009a, b; Wenzel et al. 2004).

This review aims to summarize all human studies that include a vitamin C intervention concurrent to tumour therapy or as sole therapy and examine the effect on patient-relevant endpoints.

## Methods

### Criteria for including and excluding studies in the review

Inclusion and exclusion criteria were based on the PICO model and are listed in Table 1. All studies were included that reported patient-relevant endpoints of adult cancer patients (e.g. symptoms or safety) who had received an intervention with vitamin C concurrent or after conventional tumour therapy. All cancer entities were included. Systematic reviews, meta-analyses and randomized controlled studies as well as controlled, one-arm, case–control and cohort studies were considered. Criteria for rejecting studies were primary prevention, grey literature, study populations with only precancerous conditions or children (under the age of 18) and studies that examined various diseases without separately evaluating patients with cancer. Finally, all those studies that reported no patient-relevant endpoints (but mainly laboratory parameters) were excluded. A restriction on German and English was set for the language of publication.

**Table 1:** Inclusion and exclusion criteria

PICO	Inclusion criteria	Exclusion criteria
Patient	Patients with malignancies (all entities and stages)	Patients with only precancerous conditions or carcinoma in situ
	Adult patients (age > 18)	Preclinical studies
		Study population with more than 20% children or precancerous conditions
Intervention	Every intervention with vitamin C (oral or intravenous)	
Comparison	All possible control groups (placebo, standard care, observation)	
Outcome	All patient-relevant endpoints (symptoms, safety, survival, quality of life)	No patient-centred data, for example, laboratory parameters
Others	Meta-analyses, systematic reviews, RCTs Controlled, one-arm and cohort studies	Grey literature (conference articles, abstracts, letters, ongoing studies, unpublished literature, etc.
	Language: German and English	
	Full publication	

### Study selection

A systematic search was carried out on five databases (Medline, Embase, Cochrane, Cinahl and PsycINFO) in February 2018. An update of the research took place in April 2020. For each of the databases, a complex search strategy was developed, which consisted of a combination of mesh terms, keywords and words with synonymous meaning, which were summarized in different spellings. The terms used were all related to *cancer* and *vitamin C* (Table 2). The search strategy was very comprehensive, as it was not restricted by filters to specific study types or publication forms.

**Table 2:** Search strategy

Database	Search strategy
OVID Medline	1 exp Ascorbic Acid/ or Vitamin$ c.mp. or (ascorbic$ adj1 acid$).mp. or L-ascorbic$.mp. or ascorbate$.mp. or (dehydroascorbic$ adj1 acid$).mp.;
	2 exp neoplasms/ or neoplasm$.mp or cancer$.mp. or tumo?r$.mp. or malignan$.mp. or oncolog$.mp. or carcinom$.mp. or leuk?emia.mp. or lymphom$.mp. or sarcom$.mp.;
	3 1 AND 2;
	4 limit 3 to English or limit 3 to German;
	5 (4 and humans/) or (4 not animals/)
OVID Embase	1 exp Ascorbic Acid/ or Vitamin$ c.mp. or (ascorbic$ adj1 acid$).mp. or L-ascorbic$.mp. or ascorbate$.mp. or (dehydroascorbic$ adj1 acid$).mp.;
	2 exp neoplasms/ or neoplasm$.mp or cancer$.mp. or tumo?r$.mp. or malignan$.mp. or oncolog$.mp. or carcinom$.mp. or leuk?emia.mp. or lymphom$.mp. or sarcom$.mp.;
	3 1 AND 2;
	4 limit 3 to English or limit 3 to German;
	5 (4 and humans/) or (4 not animals/)
Cochrane	#1 [mh "Ascorbic Acid"] or (ascorbic* next acid*) or L-ascorbic* or "vitamin c" or "vitamine c" or "vitamins c" or "vitamines c" or ascorbate* or (dehydroascorbic* next acid*);
	#2 [mh neoplasms] or neoplasm* or cancer? or tum*r? or malignan* or oncolog* or carcinom* or leuk*mia or lymphoma? or sarcoma?;
	#3 1 AND 2
EBSCO PsychINFO	S1 (DE "Ascorbic Acid" OR TX (ascorbic N1 acid*) OR TX L-ascorbic* OR TX "vitamin c" OR TX "vitamine c" OR "vitamins c" OR TX "vitamines c" OR TX ascorbate* OR TX (dehydroascorbic N1 acid*);
	S2 ((DE "Neoplasms" OR DE "Benign Neoplasms" OR DE "Breast Neoplasms" OR DE "Endocrine Neoplasms" OR DE "Leukemias" OR DE "Melanoma" OR DE "Metastasis" OR DE "Nervous System Neoplasms" OR DE "Terminal Cancer") OR (TX neoplasm* OR TX cancer OR TX tumo#r OR TX malignan* OR DE „oncology “ OR TX oncolog* OR TX carcinom* OR TX leuk#emia OR TX lymphoma OR TX sarcoma));
	S3 (LA German OR LA English);
	S4 S1 AND S2 AND S3
EBSCO Cinahl	S1 MH zinc or TX (zinc or zink or zn);
	S2 (MH "Ascorbic Acid") OR TX (ascorbic N1 acid*) OR TX L-ascorbic* OR TX "vitamin c" OR TX "vitamine c" OR "vitamins c" OR TX "vitamines c" OR TX ascorbate* OR TX (dehydroascorbic N1 acid*);
	S3 (LA German OR LA English);
	S4 S1 AND S2 AND S3

After all the search results had been imported into Endnote X6, duplicates were removed and a title–abstract screening was carried out by two independent assessors (CH and JH). In case of disagreement, a consensus was reached through discussion. In the next step, full texts of the remaining studies were inserted and examined again independently by both assessors. A full-text copy was also used if the title and abstract did provide enough information for screening purposes. Additionally, literature lists of all retrieved articles were checked for relevant studies.

### Assessment of risk of bias and methodological quality

#### Risk of bias

The risk of bias in the included studies was assessed using the SIGN checklist for controlled studies version 2.0 (https://www.sign.ac.uk/checklists-and-notes.html) (Fig. 2). In addition, it was investigated for all studies whether the researchers and the investigation of endpoints were blinded and whether the groups were comparable concerning demographic parameters and the examined endpoints before the beginning of the treatment.

#### Methodological quality

The included studies were rated according to the Oxford criteria (Chen et al. 2005). Further criteria with regard to the methodology were the size of the examined sample, the implementation of power analyses, the handling of missing data and drop-outs (giving reasons for drop-outs, implementation of intention-to-treat analyses), the implementation of appropriate statistical tests (e.g. checking the test requirements and avoiding multiple testing) and the comprehensive report of the endpoints (report of all recorded endpoints with documentation of the statistical parameters, such as *p* values).

### Data extraction

The evidence tables of the national guidelines on complementary and alternative medicine in oncological patients of the German guidelines program for oncology were used as a template for the data extraction (https://www.leitlinienprogramm-onkologie.de/english-language/). With regard to systematic reviews, only primary literature was extracted that met the inclusion criteria formulated for this work.

#### Study design

Systematic reviews, meta-analyses, randomized controlled studies as well as controlled, one-arm and cohort studies were included.

#### Participants

Patients were currently or had been treated with chemotherapy and radiation therapy in the past. They were characterized by the type and stage of their cancer, their age and gender.

#### Intervention

Studies that involved some form of vitamin C treatment in oncological patients were included. Type of intervention (oral or intravenous), dose, frequency and duration were extracted.

#### Comparison

Any kind of comparison was included in this review—standard care, observation groups or the administration of a placebo. Studies without comparison/control group were also included.

#### Endpoints

Primary endpoints included symptom relief and tumour development. Secondary endpoints were mortality (overall survival), the length of the progression-free interval, pain, fatigue and quality of life of the patients as well as the safety of vitamin C.

## Results

**Fig. 1:**
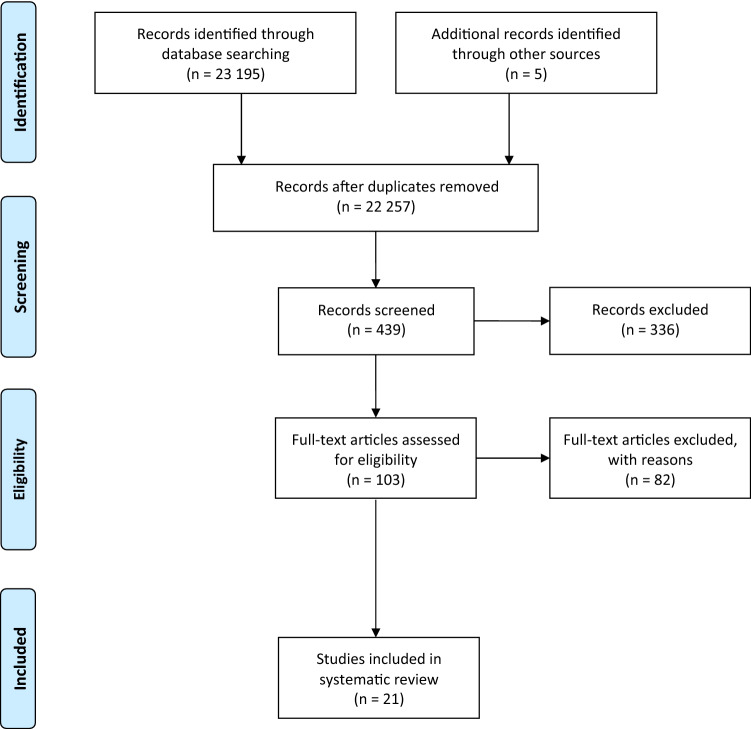
Flowchart

The systematic search resulted in a total of 23,195 hits. First of all, all duplicates were removed, leaving 22,257 publications for title–abstract screening. After that screening, 103 studies remained and were then examined in more detail. Five studies were added by hand search from reference lists. In the end, 21 publications were included in the review. We found one SR, 5 RCTs from which 3 were included in the SR and 16 studies of lower evidence. In a total of six studies, the intake of vitamin C was examined in oral form (three RCTs and three studies of lower evidence class). In the 15 remaining studies, the intervention with vitamin C was performed intravenously (two RCTs and 13 phase I/II studies). The detailed descriptions of the studies can be found in Tables 3 and 4. The flowchart of the study search and selection can be seen in Fig. 1. Frequent reasons for study exclusion were that vitamin C was administered in a multi-substance mixture, the investigation of precancerous stages or no investigation of patient-relevant endpoints. If the full text of the study could not be found, the study had to be excluded as well. Systematic reviews and meta-analyses were excluded if they only contained a very low number of studies on vitamin C or did not evaluate them individually.

**Table 3:** Characterization of the included RCTs

References	Diagnosis	Concurrent therapy	Endpoint	Dose	Results
Creagan et al. (1979)^a^	Different types of cancer	no	1. OS	10 g daily, oral	1: No significant group differences, *p* = 0.61
			2. Symptom reduction		2: No significant group differences
			3. Toxicity		3: No significant group differences
Jeon et al. (2016)	Colorectal cancer	Surgery	1. Pain	50 mg/kg (ascorbic acid 10 g/20 mL), intravenous, for 30 min	1: At rest in arm A significantly lower than in arm B: 2 h after OP (T1): A: MV = 4 (SD = 2), B: MV = 5.5 (SD = 1.5) 6 h after OP (T2): A: MV = 3.8 (SD = 2), B: MV = 5 (SD = 1.7) 24 h after OP (T3): A: MV = 3.8 (SD = 2), B: MV = 5 (SD = 1.7), T1–T3: *p* < 0.05; No group differences during coughing: T1–T3: *p* > 0.05
			2. Morphine administration after operation		2: T1: less in arm A than in arm B: A: MW = 15 mg (SD = 5), B: MW = 18 mg (SD = 5), *p* < 0.05; T2/T3: No significant group differences
			3. Fatigue		3: No significant group differences: T1–T3: *p* > 0.05
			4. Toxicity		4: Significantly less need for additional opiates in arm A: A: MV = 0.8 (SD = 0.8), B: MV = 1.4 (SD = 1), *p* = 0.00; No significant group differences with regard to post-op nausea/vomiting; Duration of hospitalization in arms A and B 1–2 days each
Liu et al. (2010)	Thyroid cancer	Radiotherapy	1. Time–activity curve of the salivary glands	100 mg every 4 h, oral, for 6 days	1: No data
			2. Dosimetry in saliva		2: No significant group differences in glandular parotid and submandibular: *p* = 0.37, 0.28
			3. Relative cumulative activity in saliva during the first 24 h		3: No significant group differences in glandular parotitis and submandibular: *p* = 0.21, 0.16
			4. Dosimetry in saliva (first 24 h)		4: No significant group differences in glandular parotitis and submandibular: *p* = 0.32, 0.24
Ma et al. (2014)^a^	Ovarian cancer	Paclitaxel/ Carboplatin therapy	1. Toxicity	15 g to 75/100 g per infusion, intravenous, for 12 months	1: No 5th-degree side effects, no significant group differences in 3rd and 4th-degree side effects; Significantly less side effects 1st and 2nd degree in arm A: *p* = 0.001, 0.03
			2. OS		2: No significant group differences—better in arm A
			3. PFS		3: No significant group differences—longer in arm A: A: 25.5 months, B: 16.75 months
Moertel et al. (1985)^a^	Colorectal cancer	no	1. OS	10 g daily, oral, for median: 2.5 months	1: No significant group differences—better OS in arm B
			2. PFS		2: No significant group differences—longer PFS in arm B: A: 2.9 months, B: 4.1 months
			3. Regression		3: Similar improvement of both arms: A: 64%, B: 65%
			4. Toxicity		4: No significant group differences, more indigestion in arm A: A: 18%, B: 6%
			5. Symptom reduction		5: No regression

*MW* mean value, *OS* overall-Survival, *PFS* progression-free-survival, *SD* standard deviation

^a^RCTs from systematic review by Jacobs et al. (2015)

**Table 4:** Characterization of the included studies with lower level of evidence

References	Diagnosis	*N*	Concurrent therapy	Endpoints	Dose	Results
Bruemmer et al. (2003)	Breast cancer, leukemia, myelodysplastic syndrome	1 182	Radio- and/or chemotherapy	1. Non-relapse mortality	0.03–12.5 g every day, oral	1: ALL and AML: At dose ≥ 500 mg daily, significant increase compared with no intake: RR (95% CI): 2.25 (1.33–3.83), *p* = 0.01
				2. Relapse		2: Mamma carcinoma: With a dose of ≥ 500 mg per day less relapse compared to no intake: RR (95% CI): 0.11 (0.02–0.89), *p* = 0.03
				3. Mortality or relapse		3: Mamma carcinoma: With a dose of ≥ 500 mg per day less relapse or mortality: RR (95% CI): 0.41 (0.17–1.02), *p* = 0.04; ALL and AML: At doses < and ≥ 500 mg daily, significant increase: < 500 mg/day: RR (95% CI): 1.40 (1.03–1.92), ≥ 500 mg/day: RR (95% CI): 1.63 (1.09–2.44), *p* = 0.01
Gunes-Bayir and Kiziltan (2015)	Different types of cancer with bone metastases	39	Radiotherapy	1. Pain	2.5 g 1 h daily, intravenous	1: Vitamin C arm: 1 × complete relief, 3 × no change, 7 × 50–80% relief, 2 × slight relief, 2 × increase in pain; Chemotherapy arm: 3 × no change, 2 × 50–80% relief, 4 × slight relief, 6 × increase in pain; Control arm: 100% increase in pain
				2. PS (ECOG: 0–5)		2: Vitamin C arm: before treatment (T0): PS = 3, increase in PS in 4 out of 15 cases after treatment (T1), otherwise no change; Chemotherapy arm: T0: PS = 4, T1: Improvement in 1 person, 14 without change; Control arm: T0: PS = 4, T1: in all 9 no change
				3. OS		3: Vitamin C arm: median: 10 months, range: 2–36 months; Chemotherapy arm: median: 2 months, range: 1–10 months, *p* < 0.001; Control arm: median: 2 months, range: 1–6 months
Hoffer et al. (2008)	Different types of cancer	24	No	1. Toxicity	0.4, 0.6, 0.9, .5 g/kg, intravenous, 3 times a week, for 4 weeks	1: Only mild side effects Grade 1 or 2
				2. Tumour response		2: No patient with an objective response of the tumour—all with progression, 2 × in 0.6 g/kg arm with stable disease
				3. QoL		3: In 0.4 g/kg arm deterioration in physical function: MV: 5.4 (SD = 4.2) vs. MV: 13.4 (SD = 1.1), *p* < 0.01; No deterioration in the higher dose groups
Hoffer et al. (2015)	Different types of cancer	14	Chemotherapy	1. Toxicity	1.5 g/kg body weight, 90 min at dose up to 90 g and 120 min at dose > 90 g, 3 × every week during chemo, otherwise every 2 days, intravenous	1: No side effects due to vitamin C
				2. Tumour response		2: 2 × very severe courses (probably unrelated to vitamin C), 6 × no change, 6 × temporary stabilization, but not long lasting, 3 × better course than expected
Kawada et al. (2014)	Non-Hodgkin lymphoma	3	Chemotherapy	1. Toxicity	15 g (0.5 g/min) on the first day, then 75 g (1 g/min), intravenous, 6 days	1: Good tolerated, no obvious side effects
Kiziltan et al. (2014)	Different types of cancer with bone metastases	11	Palliative Radiotherapy	1. Pain	2.5 g in 1 h, intravenous, 1-week intervals with 3–10 infusions	1: 48.5% reduction of pain (SD = 39.7), median: 55%, 6 × 50–100% reduction, 1 × 25% reduction, 2 × no change, 2 × aggravation
				2. PS		2: 1-year survival: 45%, 2-year survival: 17.5%
				3. Toxicity		3: 40% Grade 1 gastrointestinal toxicity, 30% Grade 1 urinary toxicity
Monti et al. (2012)	Pancreas carcinoma	9	Chemotherapy with gemcitabine or erlotinib	1. Toxicity	50 g, 75 g, 100 g per infusion, intravenous, 3 times a week, for 8 weeks	1: mild lightheadedness or nausea, 8 of 23 side effects were serious and likely due to disease progression or treatment
				2. Tumour response		2: 8 of 9 patients showed a reduction in primary tumour, 7 with stable disease, 2 with progressive disease
				3. PFS		3: MV: 89 days (SD = 77)
				4. OS		4: MV: 182 days (SD = 155)
Nielsen et al. (2017)	Prostate cancer	23	No	1. ≥ 50% PSA-level reduction	1st week: 5 g, 2nd week: 30 g, 3.-12. week: 60 g, 1 × every week, intravenous, 500 mg, oral, for 26 weeks	1: No patient achieved a 50% reduction, 75% increase in the PSA level, increase by 17 µg/L, 5 × lower PSA value than at baseline (> 2 µg/L)
				2. QoL		2: 16 × with no change, 2 × improved, 2 × worsened; changes after 12 weeks only in the physical functional level, otherwise no improvements
				3. Toxicity		3: 5 × no side effects, 4X only one side effects—in total 53 side effects (11 severe): hypertension, anaemia, pulmonary embolism
Park et al. (2009)	Leukemia, myelodysplastic syndrome	18	No	1. Toxicity	10 g-60 g/m^2^ for 2 h every day, intravenous	1: Grade 1 or 2 side effects, 1 × Grade 3 fatigue
				2. Tumour response		2: 8 × good response to therapy (1 × complete remission with incomplete recovery of the blood count, 6 × hematological reaction, 1 × hematological improvement)—8/18 (44%)
Pinkerton et al. (2017a)	Different types of cancer	17	Adjuvant therapy	1. Pain: opioid dose	1 g 2 × every day, oral, for 3 days	1: Unchanged or increased
				2. Toxicity		2: 21 side effects, that were stronger than baseline—none attributable to vitamin C
Polireddy et al. (2017)	Pancreatic cancer	12	Chemotherapy with gemcitabine	1. OS	3 × every week, intravenous	1: 50% (6/12) survived longer than a year, 8.3% (1/12) survived longer than 2 years; median: 15.1 months
				2. PFS		2: 6 × progress of disease; median: 3 months
				3. Toxicity		3: 1st Grade nausea and thirst, all others unrelated to vitamin C
Riordan et al. (2005a, b)	Different types of cancer	24	No	1. Tumour response	150, 300, 430, 570, 710 mg/kg every day, intravenous, for 8 weeks	1: 1 × stabile disease, 23 × progression
				2. Toxicity		2: 2 × Intervention discontinued due to grade 3 and 4 side effects (2 of 4 related to vitamin C), majority of side effects grade 1 or 2 (especially nausea, dry skin/mouth, oedema, fatigue)
Stephenson et al. (2013)	Different types of cancer	17	No	1. Toxicity	30, 50, 70, 90 or 110 g/m^2^ on 4 consecutive days, intravenous, for 4 weeks	1: Good safety even at high doses, mostly mild side effects and only possible through treatment; nausea and headache in all cohorts
				2. Tumour response		2: No objective tumour response, 3 × stabile course, 13 × progressing disease
				3. QoL		3: Constant over the first 2 weeks, then improvement in the 3rd and 4th week
Tareen et al. (2008)	Prostate cancer	17	No	1. PSA velocity	500 mg + 5 mg vitamin K3, 10 × every day, oral, for 12 weeks	1: Decrease in PSA velocity in 13/17 (76%): Before treatment: 1.05 to 696 ng/ml/year (median: 21.6 ng/ml/year), during treatment: -12 bis 256 ng/ml/year (median: 6.39 ng/ml/year)
				2. PSA doubling times		2: Increase in PSA doubling time in 76% Before treatment: 2.0 to 54.4 months (median: 3.12 months), during treatment: -39 bis 57.1 months (median: 7.88 months)
				3. Toxicity		3: No side effects, no discontinuation of therapy
				4. Symptoms		4: Tendency to have fewer symptoms: Before therapy: 7.9, after 12 weeks: 7.2, *p* = 0.07
				5. Pain		5. Intermittent improvement followed by decline: Before therapy: 3.2, after 6 weeks: 2.3, after12 weeks: 3.2
Vollbracht et al. (2011)	Breast cancer	125 (53/72)	Chemo- or radiotherapy	1. Intensity of symptoms	7.5 g, intravenous, 1 × every week, for at least 4 weeks	1: Mean values of the intensity in arm A smaller than in arm B, during treatment (*p* = 0.013) and after (*p* = 0.021)
				2. PS		2: Higher PS in arm A than in arm B: During treatment: arm A: 80%, arm B: 71%, *p* < 0.001; after: arm A: 87%, arm B: 78%, *p* < 0.001; ECOG: during treatment: arm A: 1.596, arm B: 2.067, *p* = 0.002; after: arm A: 1.11, arm B: 1.71, *p* < 0.001
				3. Toxicity		3: No side effects (Safety: 86.8% excellent and 13.2% good)
Welsh et al. (2013)	Pancreatic cancer	9	Chemotherapy with gemcitabine	1. Toxicity	15 g, intravenous, for 30 min, increase dose, 1 × every week, for 4 weeks	1: No severe side effect (Grade 3, 4), nausea (6×) and diarrhoea (4×), thirst and dry mouth (4×)
				2. PS		2: 6 of 9 maintained or improved PS
				3. PFS		3: 26 ± 7 weeks
				4. OS		4: 13 ± 2 months

*ALL* acute lymphocytic leukaemia, *AML* acute myelogenous leukaemia, *MV* mean value, *PS* performance status, *QoL* quality of life, *SD* standard deviation

### Patient’s characteristics of included studies

Considering all included studies, 2176 patients were assessed. Due to drop-outs because the patients withdrew from the study, rejection of vitamin C or disease progression, a total of 1961 patients could be analysed. The age ranged from 20 to 88 years across all studies. 51% of the participating patients were female and 49% male. They all suffered from various malignancies. The diagnoses included breast cancer (*n* = 243), colorectal carcinoma (*n* = 197), leukaemia (*n* = 627), thyroid carcinoma (*n* = 72), ovarian cancer (*n* = 25), pancreatic (*n* = 30) and prostate cancer (*n* = 40). 39 patients with bone metastases had different types of cancer. There were also studies in which different types of cancer were analysed together.

### Risk of bias in included studies

**Fig. 2:**
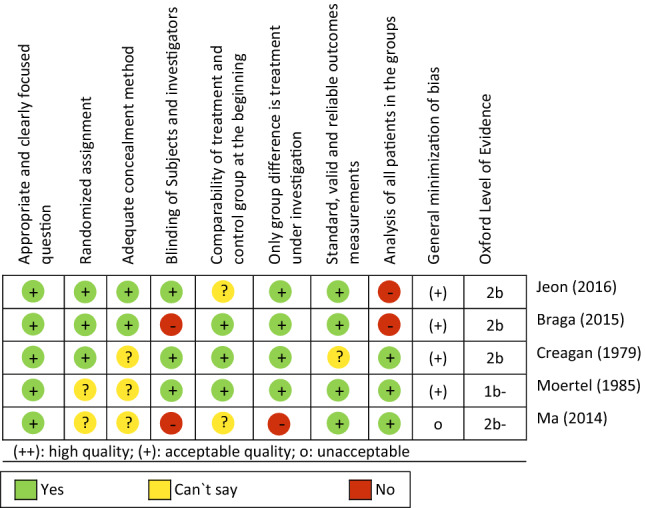
Methodical quality of the included RCTs

The detailed assessment of the risk of bias of the included RCTs can be found in Fig. 2. The process of randomization often was not explained (Creagan et al. 1979; Ma et al. 2014; Moertel et al. 1985), whereby the distribution of the patients to the examination arms remains unclear. The study by Ma et al. (2014) was not blinded and for this reason, placebo or attention effects cannot be ruled out. The risk of group differences cannot be ruled out due to a lack of baseline values (Jeon et al. 2016; Liu et al. 2010). Another common problem was reporting. In three of the six RCTs (Creagan et al. 1979; Jeon et al. 2016; Ma et al. 2014), the results were only presented graphically, without providing numerical parameters for group comparisons.

The studies of lower evidence were cohort or one-arm studies, so they mostly did not include control groups that would allow to compare to patients without vitamin C intervention. In addition, the majority of the samples were small (*n* = 39) to very small (*n* = 3), which further affects the reliability of the results. Overall, the quality of the included studies can be assessed as acceptable.

### Efficacy of complementary vitamin C therapy

#### Oral vitamin C intake in combination with tumour therapy

One RCT examined the intake of oral vitamin C in conjunction with radioiodine (Liu et al. 2010). No differences between the groups could be found. Liu et al. (2010) included 72 patients with thyroid cancer and divided them into four arms, each taking 100 mg of vitamin C every 4 h for a total of 6 days beginning 1, 5, 13 or 25 h after the start of radioiodine therapy. The influence of vitamin C on the accumulation of radioiodine in saliva or the salivary glands was measured. For the parotid gland and the submandibular gland, there were no significant differences between the treatment arms with regard to dosimetry (*p* = 0.32, 0.24) and cumulative activity in saliva (*p* = 0.21, 0.16).

Additionally, a one-arm pilot and a cohort study examined the efficacy of vitamin C in conjunction with radio- and/or chemotherapy (Bruemmer et al. 2003; Pinkerton et al. 2017b). In the study by Bruemmer et al. (2003), patients with leukaemia, breast cancer and aplastic anaemia or myelodysplastic syndrome themselves stated how much vitamin C they had consumed over the last year during therapy. The results were different. For patients with breast cancer, the risk of recurrence was reduced at a dose above 500 mg (RR (95% KI): 0.11 (0.02–0.89), *p* = 0.03), as well as the risk of mortality (RR (95% KI): 0.41 (0.17–1.02), *p* = 0.04), compared to women who did not take vitamin C. For patients with leukaemia or myelodysplastic syndrome, however, the risk of treatment-related mortality (RR (95% KI): 2.25 (1.33–3.83), *p* = 0.01) and disease progression increased significantly (< 500 mg/Tag: RR (95% KI): 1.40 (1.03–1.92), ≥ 500 mg/Tag: RR (95% KI): 1.63 (1.09–2.44), *p* = 0.01). Pinkerton et al. (2017b) analysed the effects of 1 g of vitamin C daily over three days on the opioid dose, i.e. indirectly the pain intensity of the patients. However, this remained unchanged or even increased.

#### Oral vitamin C intake as sole therapy

Two RCTs and a phase I/IIa study were found for taking vitamin C without other tumour therapy at the same time—mostly in patients with no further active cancer treatment option (Creagan et al. 1979; Moertel et al. 1985; Tareen et al. 2008). The results of the two RCTs are not significant. In the study by Creagan et al. (1979), the 123 patients with different kinds of advanced cancer were randomly assigned to receive either 10 g of vitamin C per day divided into four doses or a placebo. Taking vitamin C did not reduce symptoms, nor did it lead to an increase in survival (*p* = 0.61). Moertel et al. (1985) also gave their patients 10 g of vitamin C daily or a placebo. It was taken for a median of 2.5 months. The sample consisted of 100 patients with advanced colorectal cancer. There was no reduction in symptoms in this study either. The general lifespan and the interval at which the disease did not progress were longer in the control arm than in the vitamin C arm (vitamin C arm: 2.9 months, control arm: 4.1 months). Specific group comparisons were not calculated.

The study by Tareen et al. (2008) also showed no significant results. The prostate cancer patients received 5 g daily half the amount of vitamin C for a total of 12 weeks and had no significant symptom relief from before to after therapy (before therapy: 7.9, after therapy: 7.2, *p* = 0.07). In relation to the pain, an intermittent improvement was observed, which then turned into a worsening (before therapy: 3.2, after 6 weeks: 2.3, after 12 weeks: 3.2).

#### Interim conclusion on the oral intake of vitamin C

Considering all studies on the oral intake of vitamin C in patients with malignant diseases, there are no statistically significant effects of the intervention either alone or in combination with tumour treatment.

#### About the safety of oral vitamin C intake

In four of the seven studies on the oral intake of vitamin C, the safety of the intervention was examined (Creagan et al. 1979; Moertel et al. 1985; Pinkerton et al. 2017b; Tareen et al. 2008). None of these studies found significant vitamin C-related side effects. On the other hand, the shorter survival time with vitamin C in the study by Moertel et al. (1985) is a concern.

#### Intravenous administration of vitamin C in combination with tumour therapy

With regard to the intravenous administration of vitamin C at the same time as tumour treatment with chemo-, radiotherapy or surgery, two RCTs and eight studies with a lower level of evidence were found. In the RCT by Ma et al. (2014), 25 patients with ovarian cancer were examined. Half of them received an initial injection of 15 g vitamin C two times per week over a period of 12 months with increasing doses of up to 75–100 g per infusion in addition to chemotherapy. The control arm was treated with chemotherapy only. The disease-free interval and the general lifespan after 5 years did not differ significantly between the arms, although a trend towards longer periods was observed in the vitamin C arm (disease-free interval: vitamin C arm: 25.5 months, control arm: 16.75 months). The RCT by Jeon et al. (2016) included 97 patients with colorectal cancer. Half of the sample received 50 mg/kg vitamin C injections for 30 min during surgery and the other half received a placebo. Pain, morphine administration and fatigue of the patients were measured. The results showed that patients in the vitamin C arm experienced significantly less pain at rest, but no group differences existed during coughing (2, 6 and 12 h after surgery: at rest *p’*s < 0.05, during coughing *p’*s > 0.05). With regard to the administration of morphine, significant differences in favour of the vitamin C arm were initially noticeable, but these had disappeared 6 and 12 h after the operation (2 h after surgery: vitamin C arm: 15 mg (SD = 5 mg), control arm: 18 mg (SD = 5 mg), *p* < 0.05). There were no significant group differences concerning fatigue.

In their phase I study, Monti et al. (2012) examined patients with stage four pancreatic cancer. Either 50, 75 or 100 g of vitamin C were injected three times a week for eight weeks per cohort. The mean disease-free survival was 89 days (SD = 77), and the total lifespan was 182 days (SD = 155). Eight out of nine patients showed a reduced size of the primary tumour. Overall, the disease was described as stable in seven patients and as progressive in two. In the phase I/IIa study by Polireddy et al. (2017) in which patients with pancreatic cancer were also given vitamin C three times a week, the disease progressed in 6 of 12 patients (median: 3 months) and 6 of 12 patients survived longer than a year—1 in 12 more than 2 years (median: 15.1 months). In Welsh et al. (2013), six out of nine pancreatic cancer patients were able to maintain or improve their performance status with a vitamin injection of 15 g once a week with increasing dosage for a total of 4 weeks. The disease-free interval was 26 ± 7 weeks and the overall survival 13 ± 2 months. In patients with various other malignancies who were given 1.5 g vitamin C per kilogram of their own body weight intravenously, there were no changes in 6 of 14 patients, 6 other patients showed a temporary stabilization or a longer lasting but undulating stability of the disease and 2 of the other 14 examined a severe course, although this was not related to vitamin C. Three patients had a better course than expected (Hoffer et al. 2015). The study by Vollbracht et al. (2011) examined retrospectively the effect of vitamin C in various concurrent therapies and an intervention with 7.5 g intravenously administered vitamin C once a week for four weeks. In this cohort study, the patients with breast cancer showed a significantly better performance status both during and after treatment (*p*s < 0.001). The Eastern Cooperative of Oncology Group (ECOG) confirms this (during treatment: vitamin C arm: 1.596, control arm: 2.067, *p* = 0.002; after treatment: vitamin C arm: 1.11, control arm: 1.71, *p* < 0.001). Gunes-Bayir and Kiziltan (2015) compared retrospectively a chemotherapy group, a vitamin C group and a control group. Using 2.5 g of vitamin C given over 1 h a day, they found that patients' pain in the vitamin C arm decreased by 50–80% in seven of the 15 cases, compared to 2 of 15 in the chemotherapy arm and no patient with improvement in the control arm. Regarding the performance status, 11 patients in the vitamin C arm remained unchanged and 4 worsened. In the chemotherapy arm, 14 were without change and 1 patient with improvement. In the control arm, none showed any change. Overall survival with vitamin C was 2–36 months (median: 10 months), with chemotherapy 1–10 months (median: 2 months), and in the control arm 1–6 months (median: 2 months). In the study by Kiziltan et al. (2014), 2.5 g vitamin C was given intravenously at the same time as radiotherapy. 45% of the patients survived longer than a year, 17.5% longer than 2 years. 48.5% of the patients experienced a pain reduction.

#### Intravenous vitamin C administration as the sole therapy

No RCTs were found for intravenous intervention of vitamin C as the sole therapy, but five studies with lower evidence were found. In these studies, the main focus was on tumour response and quality of life. The phase I study by Hoffer et al. (2008) showed that the tumour did not react objectively in any patient, regardless of the dose of 0.4, 0.6, 0.9 or 1.5 g/kg vitamin C. In terms of quality of life, a deterioration in physical function was evident in the 0.4 g/kg arm. In the one-armed pilot study by Riordan et al. (2005a, b), disease progression was observed in 23 of 24 patients and stabilization in only one case. In Stephenson et al. (2013), there was also no objective reaction from the tumour. The disease progressed in 13 patients and was stable in only three. With regard to the quality of life, there was initially no change over the first two weeks, but an improvement was noted in the course of the third and fourth weeks. In the one-armed study by Nielsen et al. (2017), the quality of life remained unchanged in 16 of 23 cases, in two it improved and two developed to be worse. After 12 weeks, there was no improvement except in the area of the physical functional level. Only Park et al. (2009) reported a good response to the therapy in 44% of the cases (8/18 patients).

#### Interim conclusion on the intravenous administration of vitamin C

In summary, there is heterogeneous data. On the one hand, concurrent to tumour therapy, no significant differences in favour of intravenously administered vitamin C were found; on the other hand, it had a partially positive effect on pain and showed short-term positive effects on morphine intake. The results of the studies regarding intravenous vitamin C treatment without concurrent tumour therapy were rather negative. Vitamin C neither had an effect on the tumour nor could in prevent progression. In terms of quality of life, the data are heterogeneous.

#### About the safety of intravenous administration of vitamin C

All studies that administered vitamin C intravenously during other tumour therapies and examined the safety of the intervention demonstrated good tolerability and at most mild side effects. In Ma et al. (2014) there were generally no grade 5 side effects and grade 3 and 4 side effects were not significantly different between the two groups. In the study by Jeon et al. (2016) the vitamin C arm required significantly fewer opiates (vitamin C arm: mean = 0.8 (SD = 0.8), control arm: mean = 1.4 (SD = 1), *p* = 0.00); there were no significant group differences regarding post-operational nausea and vomiting and the length of hospitalization was comparable (1–2 days).The lower evidence studies reported dizziness, nausea, thirst, diarrhoea, dry mouth, and urinary bladder problems (Kiziltan et al. 2014; Monti et al. 2012; Polireddy et al. 2017; Welsh et al. 2013). But these were all in the first- to second-degree range. Grade 3 or 4 side effects were not found or were not due to vitamin C but to the progression of the disease or its treatment. Hoffer et al. (2008) showed only mild first- and second-degree side effects. In Riordan et al. (2005a, b), most cases were minor, as was the case with Park et al. (2009). Nielsen et al. (2017) quantified 11 out of 53 side effects as severe. Stephenson et al. (2013) came to the conclusion that vitamin C was tolerable even in higher doses and that side effects were mostly mild. The studies by Hoffer et al. (2015), Kawada et al. (2014) and Vollbracht et al. (2011) showed no side effects at all due to vitamin C.

## Discussion

### Oral vitamin C intake in combination with tumour therapy

The RCT examined the effect of orally ingested vitamin C in combination with radiotherapy on the dosimetry of the saliva and found no significant differences (Liu et al. 2010). The problem with the study was that there was no group that had not received vitamin C with which a comparison could have been made. Additionally, the size of the study arms was small. The studies of the lower level of evidence included different malignancies of the patients. The cohort study by Bruemmer et al. (2003) was not systematized, as the patients were asked to report retrospectively on how much vitamin C they had taken. The study by Pinkerton et al. (2017b) represents preliminary work that was based on a small sample and administered vitamin C over a short period of just 3 days. Overall, it is difficult to make a generalizable statement on the basis of the rather weak data presented.

### Oral vitamin C intake as sole therapy

In both RCTs on oral vitamin C intake as the sole therapy in people with advanced malignant disease, vitamin C showed neither an effect on the symptoms and overall survival (Creagan et al. 1979) nor on the disease-free interval. On the contrary, all these endpoints tended to be longer without vitamin C (Moertel et al. 1985). However, these differences were not statistically proven and neither study provided any information on the randomization process. The phase I/II study by Tareen et al. (2008) also assessed vitamin C as ineffective. The symptoms and pain were temporarily relieved, but the improvement was not lasting. As no study found reliable results in favour of oral vitamin C treatment, it must be concluded that vitamin C should not be used in patients with advanced malignancies with the aim of treating tumours or alleviating symptoms.

### Intravenous administration of vitamin C in combination with tumour therapy

No long-term effects were observed in either of the RCTs (Jeon et al. 2016; Ma et al. 2014). The study by Ma et al. (2014) is to be viewed critically as it only presents the results graphically and does not report statistical values. In addition, the groups were not blinded, the groups were not compared at the beginning and the sample was small. In the study by Jeon et al. (2016), the validity is limited due to various serious methodological deficiencies. Between the arms there may have been differences in pain perception right from the start, multiple tests were carried out and the results can only be estimated from the graphics. The eight studies at the lower level of evidence mostly showed tendencies towards a positive effect of vitamin C, as they observed reduced tumour sizes, more stable disease progression, good survival, improved or stable performance status and disease progression and pain relief (Gunes-Bayir and Kiziltan 2015; Hoffer et al., 2015; Kiziltan et al. 2014; Monti et al. 2012; Polireddy et al. 2017; Vollbracht et al. 2011; Welsh et al. 2013). However, the studies have no control groups or are retrospective. The study by Vollbracht et al. (2011) also has a bias, as the author is an employee of the manufacturing company of the vitamin C injection but did not disclose this fact in the article. Moreover, it is not clearly described how the participating centres were selected. Thus, on the basis of the presented results, general statements can hardly be made and further research would have to be carried out to confirm the positive trends.

### Intravenous administration of vitamin C as the sole therapy

With regard to the administration of vitamin C alone in intravenous form, the studies reported no reaction of the tumour regardless of the dosage (Hoffer et al., 2008; Park et al. 2009; Riordan et al. 2005a, b; Stephenson et al. 2013). Regarding the quality of life, the results related to the physical function level were contradicting (Hoffer et al. 2008; Nielsen et al. 2017). In these phase I studies, maximum dosage was 1.5 g/kg body weight or 110 g/m^2^ body surface. It should be noted here that these are exclusively low-evidence studies that were neither randomized, controlled, or blinded, and the sample sizes were small with 18–24 patients. Overall, there is concern that high intravenous doses of vitamin C can cause kidney damage. Phase 2 studies are currently ongoing.

### Vitamin C in clinical cancer care

The presented data do not provide significant indications for the assumption that vitamin C has a positive influence on the development of the tumour. While in vitro data have shown that vitamin C may lead to apoptosis of cancer cells this is not supported by the clinical data. Moreover, compared to patients without vitamin C intervention the data showed hardly any effects and vitamin C could not significantly reduce symptoms either. Thus, these findings do not give rise to the assumption that vitamin C reduces the effect of chemotherapy.

Considering safety, vitamin C as an antioxidant has been discussed critically. In the studies reported in this review, there is little clinical data on vitamin C intervention concurrently with radiotherapy. From these few studies, no worsening of outcomes has been reported (Ma et al. 2014).

Yet, a late large cohort study with patients with breast cancer reported a highly significant worse outcome for patients taking supplements with antioxidants during radio- or chemotherapy (Jung et al. 2019). Several in vitro and in vivo studies have shown that vitamin C may increase tumour growth (Chen et al. 1999; Kageyama et al. 1996; Philips et al. 2007) and lower the effect of chemotherapy and other anticancer drugs (Chen et al. 1999; Heaney et al. 2008; Kageyama et al. 1996; Llobet et al. 2008; Perrone et al. 2009a, b; Philips et al. 2007; Wenzel et al. 2004).

The reason for these seemingly contradictory data might be the different effects of vitamin C as an antioxidant in low concentration and pro-oxidant in higher concentration. So far, human studies most probably did not reach the pro-oxidant effect and it remains unclear, whether this is possible without damage to organs as the kidney.

### Limitations of our systematic review

Due to our limitation to adult studies, the results cannot be transferred to adolescents and children. In addition, only studies in English and German were included which means that the search for evidence related to vitamin C interventions in cancer patients could be expanded in further research.

## Conclusion

All in all, there are still few meaningful studies on the oral and intravenous effects of vitamin C. Some studies with lower evidence give reason to assume that vitamin could have positive effects—especially intravenous—but these have not yet been confirmed in group comparisons. Further randomized controlled studies of good methodological quality are needed to be able to make reliable and generalizable statements about the effects of vitamin C.

## Data Availability

Not applicable.
